# Anti-tumor effects of mevalonate pathway inhibition in ovarian cancer

**DOI:** 10.1186/s12885-020-07164-x

**Published:** 2020-07-29

**Authors:** Andy Göbel, Valentina M. Zinna, Stefania Dell’Endice, Nikolai Jaschke, Jan Dominik Kuhlmann, Pauline Wimberger, Tilman D. Rachner

**Affiliations:** 1 Division of Endocrinology, Diabetes, and Bone Diseases, Department of Medicine III, Technische Universität, Fetscherstraße 74, 01307 Dresden, Germany; 2 German Cancer Consortium (DKTK), Partner Site Dresden and German Cancer Research Center (DKFZ), Heidelberg, Germany; 3 Institute for Research in Biomedicine (IRB Barcelona), Barcelona Institute of Science and Technology, Barcelona, Spain; 4 Center for Healthy Aging, Technische Universität Dresden, Dresden, Germany; 5 Department of Internal Medicine I, Gastroenterology, Hepatology, Endocrinology and Metabolism, Medical University of Innsbruck, Innsbruck, Austria; 6 Department of Gynecology and Obstetrics, Medical Faculty and University Hospital Carl Gustav Carus, Technische Universität Dresden, Dresden, Germany; 7 National Center for Tumor Diseases (NCT), Dresden, Germany: German Cancer Research Center (DKFZ), Heidelberg, Germany; Faculty of Medicine and University Hospital Carl Gustav Carus, Technische Universität Dresden, Dresden, Germany; Helmholtz-Zentrum Dresden - Rossendorf (HZDR), Dresden, Germany

**Keywords:** Ovarian cancer, Statins, Amino-bisphosphonates, Apoptosis, Mevalonate pathway

## Abstract

**Background:**

Ovarian cancer remains the most fatal gynecological malignancy. Current therapeutic options are limited due to late diagnosis in the majority of the cases, metastatic spread to the peritoneal cavity and the onset of chemo-resistance. Thus, novel therapeutic approaches are required. Statins and amino-bisphosphonates are inhibitors of the mevalonate pathway, which is a fundamental pathway of cellular metabolism, essential for cholesterol production and posttranslational protein farnesylation and geranylgeranylation. While this pathway has emerged as a promising treatment target in several human malignancies, its potential as a therapeutic approach in ovarian cancer is still not fully understood.

**Methods:**

Human ovarian cancer cell lines (IGROV-1, A2780, A2780cis) were treated with increasing concentrations (0.5-100 μM) of statins (simvastatin, atorvastatin, rosuvastatin) and zoledronic acid. Effects on cell vitality and apoptosis were assessed using Cell Titer Blue®, Caspase 3/7 Glo®, clonogenic assays as well as cleaved poly (ADP-ribose) polymerase (cPARP) detection. The inhibition of the mevalonate pathway was confirmed using Western Blot of unprenylated Ras and Rap1a proteins. Quantitative real-time PCR and ELISA were used to analyze modulations on several key regulators of ovarian cancer tumorigenesis.

**Results:**

The treatment of IGROV-1 and A2780 cells with statins and zoledronic acid reduced vitality (by up to 80%; *p* < 0.001) and induced apoptosis by up to 8-folds (*p* < 0.001) in a dose-dependent fashion. Rescue experiments using farnesyl pyrophosphate or geranylgeranyl pyrophosphate evidenced that blocked geranylgeranylation is the major underlying mechanism of the pro-apoptotic effects. Gene expression of the tumor-promoting cytokines and mediators, such as transforming growth factor (TGF)-β1, vascular endothelial growth factor (VEGF), interleukin (IL)-8, and IL-6 were significantly suppressed by statins and zoledronic acid by up to 90% (*p* < 0.001). For all readouts, simvastatin was most potent of all agents used. Cisplatin-resistant A2780cis cells showed a relative resistance to statins and zoledronic acid. However, similar to the effects in A2780 cells, simvastatin and zoledronic acid significantly induced caspase 3/7 activation (6-folds; *p* < 0.001).

**Conclusion:**

Our in vitro findings point to promising anti-tumor effects of statins and zoledronic acid in ovarian cancer and warrant additional validation in preclinical and clinical settings.

## Background

Ovarian cancer is the leading cause of death from gynecological malignancies in women [1]. The survival rate of affected patients is comparably poor, especially due to the diagnosis at an advanced stage in the vast majority of the cases [2–4]. A consistent number of patients present few clinical symptoms, which are also characteristic of various gastrointestinal, abdominal and urinary conditions [5, 6]. In many cases, the late diagnosis leads to a clinical picture that includes intraperitoneal dissemination of cancer cells [6]. Here, increased permeability of the peritoneal membrane and its associated vasculature leads to massive fluid accumulation (i.e. ascites) within the peritoneal cavity [7, 8]. This process is sustained and accelerated by inflammatory cytokines, chemokines and growth factors secreted by cancer cells and additional cellular components of the tumor microenvironment, such as lymphocytes and tumor-associated macrophages (TAMs) [4, 8, 9]. The resulting pro-inflammatory microenvironment supports the malignant invasive growth of the tumor and drives morbidity and mortality of affected patients [4]. Furthermore, ascites is associated with chemoresistance, recurrence, and an overall poor prognosis [4, 8]. Current therapeutic options in ovarian cancer management depend on the diagnosed stage and include radical cytoreductive surgery, which improves remission rates and results in a longer duration of tumor-free survival in over 90% of early-diagnosed cases [10]. Adjuvant chemotherapy is more commonly administered in advanced ovarian cancer using carboplatinum and paclitaxel therapy which precedes and follows surgical cytoreduction [11, 12]. The anti-angiogenic drug bevacizumab was recently approved as an additional first-line therapy [13, 14]. Bevacizumab is administered in combination with the platinum-based chemotherapy and afterwards as maintenance monotherapy for a total of 15 months as used in the GOG-0218 trial [13–15]). In addition, poly (ADP-ribose) polymerase (PARP) inhibitors have been approved in ovarian cancer [16]. Here, olaparib is approved as maintenance therapy after initial platinum-based chemotherapy for *BRCA1/2* mutation carriers [17]. In so-called platinum-sensitive ovarian cancer relapse PARP inhibitors are approved as maintenance therapy after response to platinum-based chemotherapy independent on the *BRCA* mutation status [18–21]. Major challenges in the management of ovarian malignancies are chemo-resistance to platinum-based therapy, metastases and disease recurrence [22]. Although patients with resistant tumors eventually respond to second-line therapies, a large proportion of them experience short disease-free survival [23]. Therefore, novel therapeutic options, especially for patients with chemo-resistant ovarian cancer, are urgently needed.

The mevalonate pathway is responsible for the biosynthesis of sterol and non-sterol isoprenoids, thereby playing a central role in cellular metabolism [24, 25]. Moreover, it is important for the post-translational modifications of proteins, specifically by providing farnesyl pyrophosphate (FPP) or geranylgeranyl pyrophosphate (GGPP) for farnesylation and geranylgeranylation, referred to as protein prenylation [26]. Due to their central role in regulating cellular signaling processes, Rho-GTPases such as the Ras superfamily are among the best-studied prenylated proteins [27, 28]. The dysregulation and involvement of mevalonate pathway enzymes and products such as cholesterol in human malignancies has been shown for several tumor entities including ovarian cancer [25, 29–33]. Cholesterol can also be converted to 27-hydroxycholesterol by cytochrome P450 oxidase CYP27A1, which is expressed by both tumor cells and TAMs. CYP27A1 expression and 27-hydroxycholesterol promote tumor growth and are associated with reduced progression-free survival in breast and ovarian cancer [34, 35]. In addition, Rho-GTPases are implicated in ovarian cancer tumorigenesis and platinum resistance [36, 37]. Two major classes of drugs inhibit the mevalonate pathway at different levels: statins and amino-bisphosphonates (N-BP). Statins are inhibitors of the 3-hydroxy-3-methylglutaryl-CoA reductase (HMGCR), the rate-limiting enzyme of the mevalonate pathway. They reduce cholesterol production and mediate the increased uptake of extracellular cholesterol by low density lipoprotein receptors [38, 39]. Statins have evolved as a standard care for treating high cholesterol levels in patients [39, 40]. N-BP are inhibitors of the farnesyl diphosphate synthase and induce apoptosis in bone-resorbing osteoclasts [41, 42]. Therefore, N-BP are clinically used in osteoporosis or osteolytic bone metastases secondary to breast and prostate cancer [41, 42]. Both classes of mevalonate pathway inhibitors show a multitude of pleiotropic antitumor effects, ranging from induction of apoptosis, inhibition of migration, metastasis and invasion, as well as modulation of tumor-promoting signaling molecules or the immune system [25, 43–46]. In this study, we aimed at investigating the in vitro anti-tumor effects of several statins and the N-BP zoledronic acid in different human ovarian cancer cell lines.

## Methods

### Cancer cell lines and cell culture

Human ovarian cancer cell lines A2780 and A2780cis were obtained from Sigma-Aldrich (Munich, Germany) in 2013 (catalogue numbers # 93112519 and #93112517). The IGROV-1 cell line was a kind gift of Dr. Jean Benard (Villejuif, France) in 2014. All cell lines have been authenticated by STR profiling at the DSMZ (Braunschweig, Germany) and tested for mycoplasma contamination by a standard PCR test. IGROV-1, A2780, and A2780cis were cultured in RPMI 1640 medium (Gibco®, Life Technologies, Darmstadt, Germany), supplemented with 10% fetal calf serum (FCS; Biochrom, Berlin, Germany) and 1% penicillin/streptomycin (Biochrom, Berlin, Germany). IGROV-1 cells are cisplatin-sensitive cells, derived from a stage III ovarian carcinoma patient [47]. A2780cis cells were originally established as a cisplatin-resistant subclone of parental cisplatin-sensitive A2780 cells by chronic exposure to increasing cisplatin concentrations [48]. All cell lines were incubated in a humidified atmosphere at 37 °C under 5% CO_2_. To maintain chemo-resistance, A2780cis cells were supplemented with 1 μM cisplatin every 2–3 passages.

### Reagents and antibodies

Cells were treated with simvastatin (SIM), rosuvastatin calcium (ROSU), atorvastatin calcium salt trihydrate (ATO), zoledronic acid (ZOL), farnesyl pyrophosphate (FPP), and/or geranylgeranyl pyrophosphate (GGPP). Stocks were prepared in dimethyl sulfoxide (DMSO), ddH_2_O or were already delivered as solutions. All reagents were purchased from Sigma-Aldrich (Munich, Germany), except for ROSU (SelleckChem, Munich, Germany). Primary antibodies for Western Blot analyses were: anti-Rap1A (sc-1482; recognizes the ungeranylgeranylated Rap1a [49]), anti-Ras (#610001) from BD Biosciences (Heidelberg, Germany), and anti-cleaved PARP (#9541) from Cell Signaling Technology, Inc. (Beverly, MA, USA). For GAPDH, the anti-GAPDH (sc-25,778) from Santa Cruz (Heidelberg, Germany) and anti-GAPDH (#5G4) from HyTest Ltd. (Turku, Finland) were used. Secondary horseradish peroxidase (HRP)-conjugated antibodies were anti-rabbit IgG (HAF008), anti-mouse IgG (HAF007), and anti-goat IgG (HAF109) from R&D Systems, Inc. (Minneapolis, MN, USA).

### Vitality, apoptosis, and clonogenic assays of cancer cells

Cell viability was assessed using the CellTiterBlue® assay (Promega, Mannheim, Germany) according to the manufacturer’s protocol. A Caspase 3/7 Glo® assay (Promega) and the detection of the cleaved fragment of poly (ADP-ribose) polymerase (PARP) were performed to detect apoptosis. Caspase 3/7 Glo® and CellTiterBlue® measurements were completed using the FluoStar Omega (BMG labtech, Jena, Germany). For the clonogenic assay [50], 200 vital IGROV-1 cells were seeded in each cavity of 6-well plates and treated with statins and zoledronic acid, according to the indicated concentrations. Plates were incubated at 37 °C and 5% CO_2_ for 9–10 days, until a sufficient number of colonies was reached, but before individual colonies started to fuse. A colony was defined as containing at least 50 cells. Colonies were washed with PBS, fixed with 10% paraformaldehyde for 30 min and stained with 0.02% crystal violet in 2% ethanol at room temperature (RT) for 15 min. Plates were washed with tap water and dried at RT. Elution of the crystal violet staining was done with 10% SDS and absorbance was measured at 595 nm.

### RNA isolation, reverse transcription, and quantitative real-time polymerase chain reaction (qRT-PCR)

The analysis of gene expression was performed as previously described [51]. RNA isolation was performed using the High Pure RNA Isolation Kit (Roche; Mannheim, Germany), according to the manufacturer’s protocol. RNA samples were quantified using Nanodrop (ND-1000, Thermo Fisher Scientific, Erlangen, Germany). Reverse transcription of 500 ng RNA was performed using SuperScript II reverse transcriptase and RNaseOUT™ Recombinant Ribonuclease inhibitor (all from Invitrogen, Karlsruhe, Germany). The cDNA samples were analyzed for gene expression by qRT-PCR, using a Power SYBR® green-based PCR MasterMix (Applied Biosystems, Darmstadt, Germany), according to a standard protocol with the 7500 Fast Real-Time PCR System (Applied Biosystems, Carlsbad, CA, USA). The primer sequences (Sigma-Aldrich, Hamburg, Germany) that were used are listed in Table 1. The results were examined using the ΔΔCT method and are shown as changes of gene expression relative to the GAPDH housekeeping gene.

**Table 1:** Primers used in the study

Genes	Sequences (5′-3′)
***BCL-2***	F: TGTGTGTGGAGAGCGTCAAC R: GACAGCCAGGAGAAATCAAAC
***GAPDH***	F: AGCCACATCGCTCAGACAC R: GCCCAATACGACCAAATCC
***IL6***	F: TACCCCCAGGAGAAGATTCC R: TTTTCTGCCAGTGCCTCTTT
***IL-8***	F: CTGGACCCCAAGGAAAACTG R: TTCTCAGCCCTCTTCAAAAAC
***SVV***	F: GAACTGGCCCTTCTTGGAG R: AAGTCTGGCTCGTTCTCAGTG
***TGFβ1***	F: TGCTAATGGTGGAAACCCACAACG R: TCTCGGAGCTCTGATGTGTTGAAG
***TNFA***	F: CTCCTCACCCACACCATCAG R: GGAAGACCCCTCCCAGATAG
***VEGF***	F: GTGATGATTCTGCCCTCCTC R: CCTTGCTGCTCTACCTCCAC

*BCL-2* B-cell lymphoma 2, *GAPDH* glyceraldehyde 3-phosphate dehydrogenase, *IL6* interleukin 6, *IL8* interleukin 8, *SVV* survivin, *TGFβ1* transforming growth factor β1, *TNFA* tumor necrosis factor α, *VEGF* vascular endothelial growth factor

### Immunoblotting

Western blot analyses were performed as previously described [52]. Total protein was isolated from treated cells by using a sodium dodecyl sulfate (SDS)-based lysis buffer (20 mM Tris/HCl pH 7.4; 1% SDS; protease inhibitor cocktail (Roche)). Ten to 20 μg of protein were loaded on a 10–12% SDS polyacrylamide gel electrophoresis (SDS-PAGE). Proteins were blotted on nitrocellulose membranes (0.2 μm) and blocked using 5% bovine serum albumin (BSA) or 5% nonfat dry milk in Tris-buffered saline with 1% Tween-20 (TBS-T) at RT for at least 1 h. After washing in 1x TBS-T, membranes were incubated with primary antibody in blocking buffer at 4 °C overnight. Subsequently, membranes were washed in 1x TBS-T and incubated with HRP-conjugated secondary antibodies at RT for 1 h. The luminescent ECL detection kit (Pierce, Thermo Fisher Scientific, Schwerte, Germany) was used for visualization of the proteins. Chemiluminescence was detected using MF-ChemiBIs 3.2 imager (Bio-Imaging Systems, Germany) and GelCapture 7.0.18 software (DNR Bio-Imaging System Ltd.). Original blots were cropped for the arrangement of the final figures and are included in the supplementary (Suppl. Figs. 5, 6, 7, 8).

### Enzyme-linked immunosorbent assay (ELISA)

A human IL-6 ELISA kit (Peprotech, Hamburg, Germany) was used to assess the secreted levels of IL-6 in the supernatants of IGROV-1 cells. Briefly, 96-well ELISA plates (biomat srl, Rovereto, Italy) were coated with capture antibody (1 μg/ml) against IL-6 and incubated overnight at 4 °C. Wells were washed (0.05% Tween-20 in PBS) and blocked with 1% BSA in PBS. After washing, ELISA plates were coated with supernatants of IGROV-1 cells (1:20 in 0.05% Tween-20 and 0.1% BSA in PBS) and incubated for 1 h at RT. To detect cytokines bound to the capture antibody, ELISA plates were washed again and subsequently coated with a detection antibody (0.25 μg/ml) and Avidin-HRP-conjugate (1:2000). The color development after adding ABTS substrate (Sigma-Aldrich, Hamburg, Germany) was detected using the FluoStar Omega (BMG labtech) at 405 nm, with wavelength corrected at 650 nm.

### Statistical analyses and software

Results are presented as means ± standard error of the mean (SEM), from experiments in technical duplicates repeated at least three times with biological replicates. Outliers were determined via Grubb’s test. Group analyses were performed using one-way analysis of variance (ANOVA) by GraphPad Prism 6.07 (GraphPad, La Jolla, CA, USA). For direct comparisons between A2780 and A2780cis cells, a two-way ANOVA with a Bonferroni post-test was performed. *P*-values < 0.05 were considered statistically significant.

## Results

### Statins and zoledronic acid inhibit the mevalonate pathway and suppress vitality in IGROV-1 and A2780 cells

**Fig. 1:**
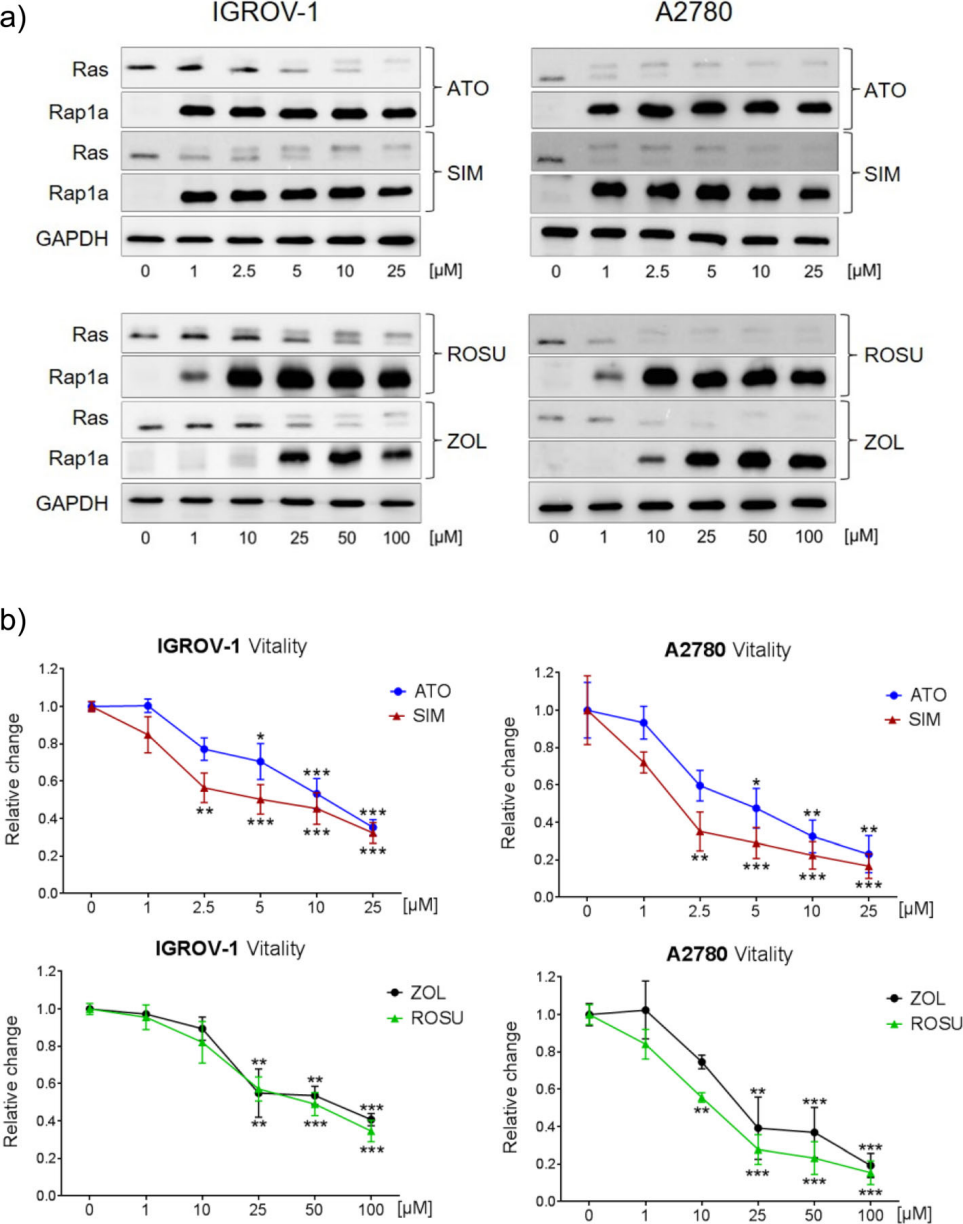
The inhibition of the mevalonate pathway suppresses cell vitality in human IGROV-1 and A2780 ovarian cancer cells. **a** IGROV-1 and A2780 cells were treated for 48 h and 24 h, respectively, with increasing concentrations of atorvastatin (ATO), simvastatin (SIM), rosuvastatin (ROSU) or zoledronic acid (ZOL). The inhibition of the mevalonate pathway was assessed by detection of Ras-related protein 1 (Rap1a, ungeranylated form is detected) and unfarnesylated Rat sarcoma (Ras; upper band) using Western blot. Glyceraldehyde-3-phosphate dehydrogenase (GAPDH) was used as loading control. The figures show representative blots which were cropped from original images. Full-length blots are presented in Suppl. Fig. 5. Images were detected using GelCapture 7.0.18 software. **b** IGROV-1 and A2780 cells were treated with increasing concentrations of ATO, SIM, ROSU, or ZOL for 48 h. Cell vitality was assessed by CellTiterBlue® assay. Data are shown as mean ± SEM of at least three individual experiments. (*p < 0.05; **p < 0.01; ***p < 0.001)

IGROV-1 and A2780 ovarian cancer cell lines were treated with increasing concentrations of atorvastatin, simvastatin, rosuvastatin, or zoledronic acid. IGROV-1 cells were treated for 48 h while A2780 cells were treated for 24 h due to a higher sensitivity. The effective inhibition of the mevalonate pathway was verified by the accumulation of ungeranylgeranylated Rap1a and unfarnesylated Ras (Fig. 1a). While unprenylated Rap1a appeared upon treatments as a single band, unprenylated Ras was distinguishable by a second smaller band above the prenylated protein. At a concentration of 1 μM, atorvastatin and simvastatin were already sufficient to block the mevalonate pathway, indicated by a strong accumulation of ungeranylgeranylated Rap1a (Fig. 1a). The vitality of IGROV-1 cells was reduced in a dose-dependent fashion with a maximal loss of 60% at a concentration of 25 μM simvastatin/atorvastatin (Fig. 1b; *p* < 0.001). IGROV-1 cells were insensitive to 1–10 μM rosuvastatin/zoledronic acid, but a significant loss of vitality was achieved by 25–100 μM (Fig. 1b; *p* < 0.001). Interestingly, the clonogenic potential of IGROV-1 cells was already significantly suppressed by 0.5 μM simvastatin (− 55% number of colonies; Suppl. Fig. 1; *p* < 0.001). Compared to IGROV-1, A2780 cells responded with a higher sensitivity to any of the substances, with a reduction of cell vitality by up to 80% using the highest concentrations after 48 h (Fig. 1b; *p* < 0.001). In both cell lines, simvastatin was the most potent of the used statins, with a significant reduction of cell vitality at 2.5 μM (*p* < 0.001).

### Inhibition of the mevalonate pathway induces apoptosis in IGROV-1 and A2780 cells

**Fig. 2:**
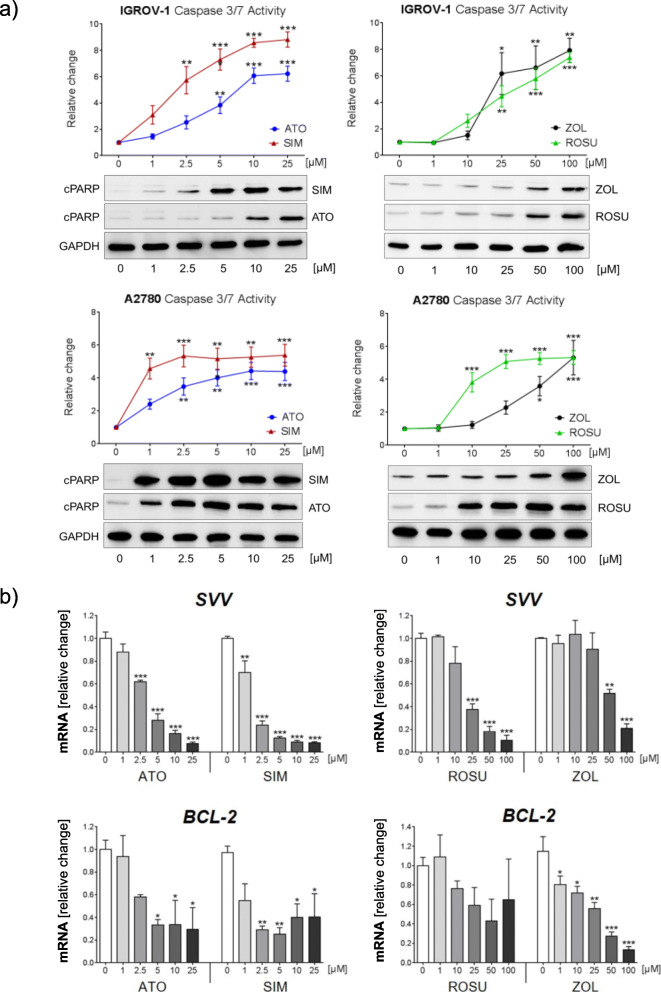
The inhibition of the mevalonate pathway induces apoptosis in human IGROV-1 and A2780 ovarian cancer cells. **a** Anti-tumor effects mediated by atorvastatin (ATO), simvastatin (SIM), rosuvastatin (ROSU) or zoledronic acid (ZOL) were assessed by the Caspase 3/7 Glo® assay after treatment of IGROV-1 and A2780 cells for 48 h. Induction of apoptosis was further confirmed using Western blot based detection of cleaved poly (ADP-ribose) polymerase (cPARP) 48 h after treatment of IGROV-1 cells and 24 h after treatment of A2780 cells, respectively. The equal protein loading is shown by detection of glyceraldehyde-3-phosphate dehydrogenase (GAPDH). The figures show representative blots which were cropped from original images. Full-length blots are presented in Suppl. Fig. 6. Images were detected using GelCapture 7.0.18 software. **b** Analysis of the expression of the anti-apoptotic genes *survivin* (*SVV*) and *B-cell lymphoma 2* (*BCL-2*) by quantitative real-time-PCR 48 h after treatment of IGROV-1 cells with ATO, SIM, ROSU and ZOL. Data are shown as mean ± SEM of at least three individual experiments. (*p < 0.05; **p < 0.01; ***p < 0.001)

Next, we assessed activation of caspases 3 and 7 after treatment of IGROV-1 and A2780 cells for 48 h with the same concentrations of statins and zoledronic acid. A significant induction of caspases 3/7 activity, by up to 6-folds and up to 8-folds, was observed with increasing concentrations of any statin and zoledronic acid in A2780 and IGROV-1, respectively (Fig. 2a; *p* < 0.001). Again, A2780 cells appeared to be more sensitive to statins compared to IGROV-1 cells as a significant activation of caspases 3/7 was obtained using 1 μM simvastatin or 10 μM rosuvastatin (*p* < 0.01). In both cell lines, simvastatin was the most potent of the tested statins and induced a 3-folds increase of caspases activity in IGROV-1 cells and a 4.5-folds increase in A2780 at the lowest concentration of 1 μM (Fig. 2a). Higher concentrations of zoledronic acid (> 25 μM) were necessary to achieve significant results. Apoptosis was confirmed by assessment of cleaved (poly-ADP) ribose polymerase (cPARP) in both cell lines (Fig. 2a). A2780 cells were more sensitive to the treatments with a strong accumulation of cPARP already 24 h after treatment. Notably, the treatment of IGROV-1 cells also significantly suppressed the gene expression of the two anti-apoptotic genes *B-cell lymphoma 2* (*Bcl-2*) and *survivin* (*Svv*). Whereas this suppressive effect was more pronounced on *Svv* using any statin (by up to − 90% gene expression; Fig. 2b; *p* < 0.001), zoledronic acid had a stronger effect on *Bcl-2* expression (− 85% gene expression; Fig. 2b; *p* < 0.001). Rosuvastatin was not able to significantly reduce *Bcl-2* expression. *Bcl-2* levels in A2780 cells were not affected by any of the mevalonate pathway inhibitors (Suppl. Fig. 2). By contrast, *Svv* expression was decreased by all agents, with simvastatin being the most potent one, which suppressed *Svv* gene expression by 50% at a concentration of 2.5 μM (Suppl. Fig. 2; *p* < 0.01).

### The anti-tumor effects of statins and zoledronic acid in IGROV-1 and A2780 cells are mediated by an inhibited geranylgeranylation

**Fig. 3:**
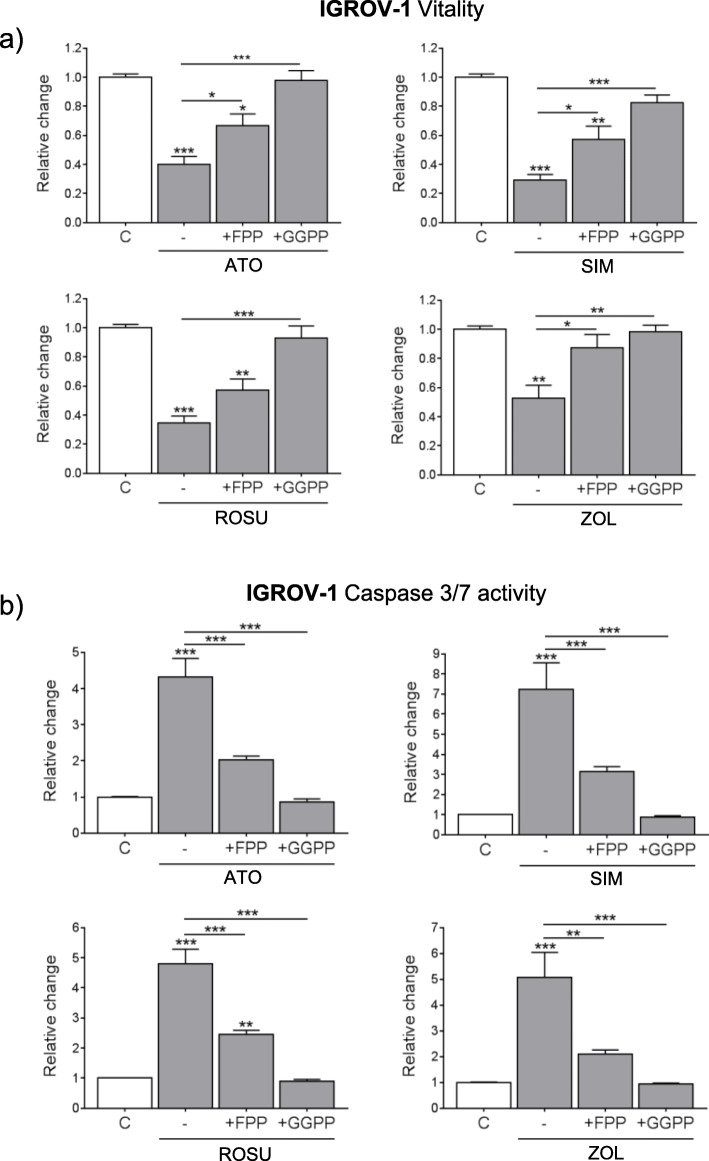
The anti-tumor effects by simvastatin (SIM), atorvastatin (ATO), rosuvastatin (ROSU) and zoledronic acid (ZOL) in IGROV-1 cells are mediated by inhibited geranylgeranylation. IGROV-1 cells were treated with ATO (10 μM), SIM (10 μM), ROSU (50 μM) or ZOL (50 μM), and supplemented with 10 μM of either farnesyl pyrophosphate (FPP) or geranylgeranyl pyrophosphate (GGPP) for 48 h. **a** Cell vitality was assessed by CellTiterBlue® assay. **b** Apoptosis was measured by the Caspase 3/7 Glo® assay. Data are shown as mean ± SEM of at least three individual experiments. (*p < 0.05; **p < 0.01; ***p < 0.001 vs. respective control (C) or vs. respective treatment (−))

The mevalonate pathway is essential for farnesylation and geranylgeranylation of proteins by the production of FPP and GGPP. To distinguish between the role of one or the other prenylation route in the anti-tumor effects of statins and zoledronic acid in ovarian cancer, rescue experiments were performed. IGROV-1 cells were supplemented with FPP or GGPP to specifically rescue farnesylation or geranylgeranylation during mevalonate pathway inhibition. The individual efficacy of both supplements to recover farnesylation or geranylgeranylation upon mevalonate pathway inhibition was proven for the treatments with simvastatin or zoledronic acid (Suppl. Fig. 3a). Only GGPP was able to recover suppressed cell vitality (Fig. 3a) or activation of caspases 3/7 (Fig. 3b) upon the treatments with any statin and zoledronic acid (*p* < 0.001). FPP only partially rescued the observed effects. Similar observations were made in A2780 cells, where loss of vitality by mevalonate pathway inhibition was almost fully rescued by GGPP but not FPP supplementation (Suppl. Fig. 3b; *p* < 0.001). Hence, blocked geranylgeranylation appeared more critical in mediating the anti-tumor effects by mevalonate pathway inhibition in IGROV-1 cells.

### Statins and zoledronic acid suppress pro-inflammatory cytokines in IGROV-1 cells

**Fig. 4:**
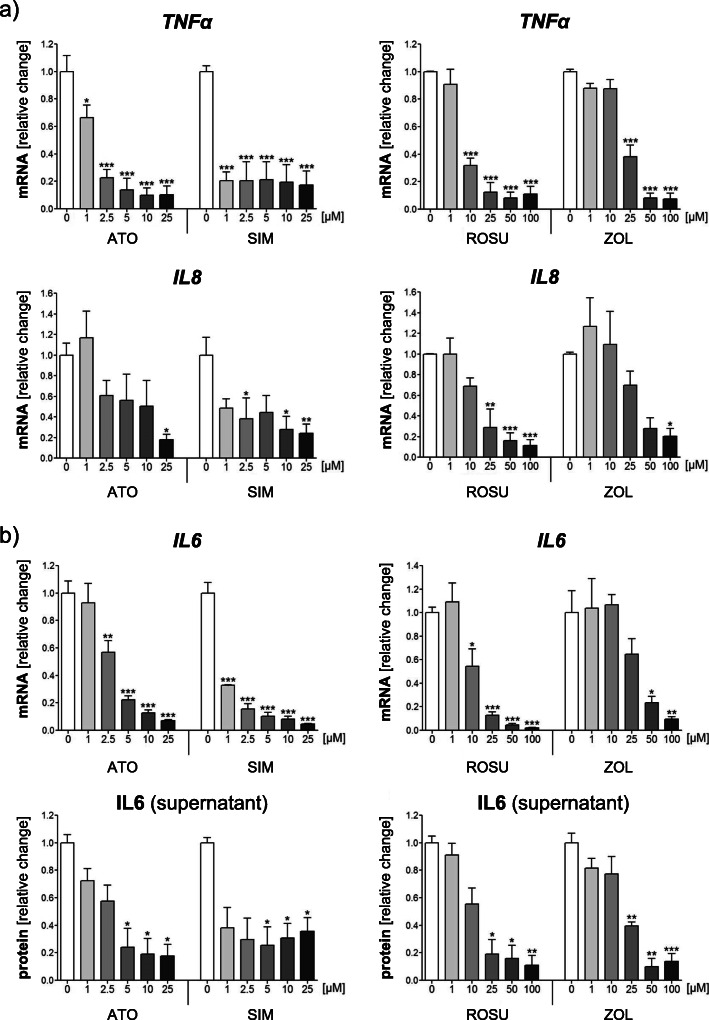
Statins and zoledronic acid (ZOL) suppress gene and protein expression of pro-inflammatory cytokines in IGROV-1 cells. IGROV-1 cells were treated with increasing concentrations of atorvastatin (ATO), simvastatin (SIM), rosuvastatin (ROSU) or ZOL for 24 h. Expression of *tumor necrosis factor (TNF)α* and *interleukin* (*IL)8* was assessed by quantitative real-time-PCR (**a**). Expression of *IL6* was assessed by quantitative real-time-PCR after 24 h of treatments (**b**). Supernatants of treated IGROV-1 cells were collected after 48 h of treatments and analyzed for IL-6 protein levels by human IL-6 ELISA (**b.**) Data are shown as mean ± SEM of at least three individual experiments. (*p < 0.05; **p < 0.01; ***p < 0.001 vs. respective control (0 μM))

Several mediators support the growth, progression, and metastasis of human ovarian cancer cells. The pro-inflammatory cytokines IL-6, IL-8 and TNF-α are commonly present in the ascites of ovarian cancer patients [53, 54]. Therefore, the expression of these tumor-promoting genes was assessed in IGROV-1 cells 24 h after treatments. All statins and zoledronic acid significantly suppressed gene expression of *TNFα* and *IL8* (Fig. 4a; *p* < 0.001). For *TNFα*, a *s*ignificant reduction was already achieved upon 1 μM of simvastatin or atorvastatin, whereas 10–25 μM rosuvastatin or zoledronic acid were necessary to obtain comparable results (Fig. 4a). In general, higher concentrations of all agents were needed to significantly block *IL8* expression. Moreover, statins and zoledronic acid dose-dependently and significantly inhibited gene expression of *IL6* (− 95%; Fig. 4b; *p* < 0.001). Again, a strong inhibitory effect was already seen with 1 μM simvastatin. These results were reflected by IL-6 protein levels measured in the supernatants of IGROV-1 treated cells. Lower concentrations of atorvastatin and simvastatin (5 μM) achieved a significant decrease of IL-6 protein by 80%, whereas only higher levels of rosuvastatin and zoledronic acid (25 μM) led to comparable results (Fig. 4b; *p* < 0.001).

### Statins and zoledronic acid suppress tumor-promoting regulators in IGROV-1 and A2780 cells

**Fig. 5:**
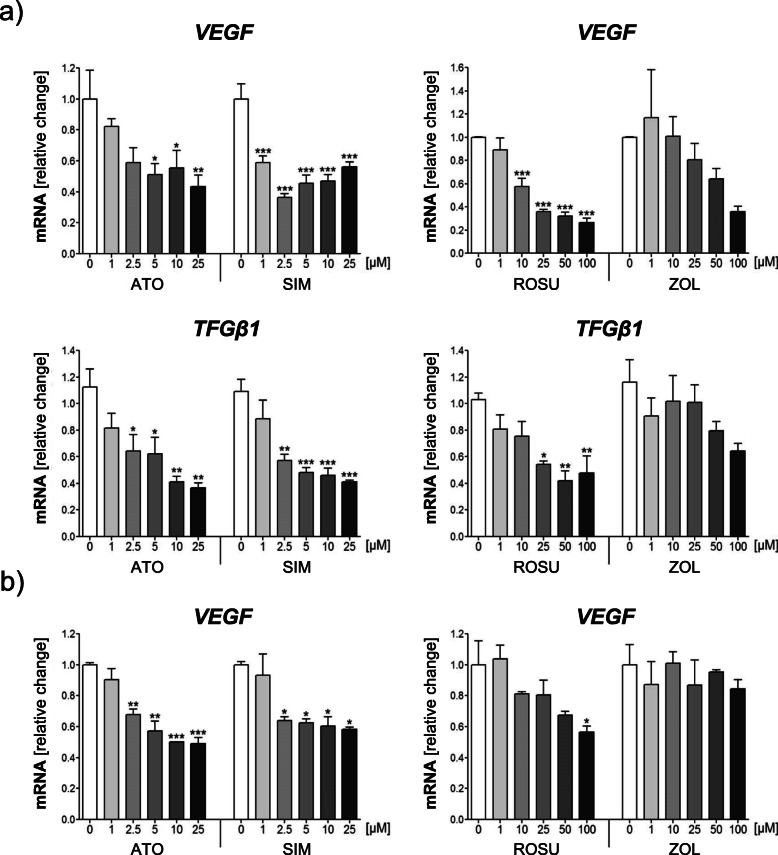
Statins reduce the expression of *vascular endothelial growth factor* (*VEGF*) and *transforming growth factor* (*TGF*)-*β1* in IGROV-1 and A2780 cells. IGROV-1 (**a**) and A2780 (**b**) cells were treated with increasing concentrations of atorvastatin (ATO), simvastatin (SIM), rosuvastatin (ROSU) or zoledronic acid (ZOL). Expression of *VEGF* (24 h) and *TGFβ1* (48 h) was assessed by quantitative real-time-PCR. Data are shown as mean ± SEM of at least three individual experiments. (*p < 0.05; **p < 0.01; ***p < 0.001 vs. respective control (0 μM))

An inflammatory microenvironment in ovarian cancer accelerates the dissemination of tumor cells and metastases. These events are facilitated by an increased vasculature permeability and by epithelial-mesenchymal transition. In this regard, the pro-angiogenic vascular endothelial growth factor (VEGF) and the pro-migratory transforming growth factor (TGF)-β1 are considered key elements contributing to tumor progression [9, 55]. Expression of *VEGF* was measured when IGROV-1 and A2780 cells were treated for 24 h, whereas *TGFβ1* expression was analyzed after 48 h of treatments in IGROV-1 cells, since mild or no effects were observed after 24 h (data not shown). All statins significantly reduced *VEGF* and *TGFβ1* gene expression in IGROV-1 cells by up to 60% (Fig. 5a; *p* < 0.001). Again, simvastatin was the most effective statin, leading to a 40% reduction of *VEGF* at 1 μM (*p* < 0.001), and of *TGFβ1* at 2.5 μM (*p* < 0.01). The effects were not enhanced by higher concentrations. Rosuvastatin proved to be effective in lowering *VEGF* expression when used at 10 μM (*p* < 0.001), and mediated a significant *TGFβ1* reduction only at the highest concentrations (*p* < 0.01). Zoledronic acid failed to induce a significant response. *VEGF* expression was also seen to be significantly decreased in A2780 cells upon statins, but not upon treatment with zoledronic acid (− 50%; Fig. 5b; *p* < 0.001).

### Simvastatin induces apoptosis in cisplatin-resistant A2780cis cells

**Fig. 6:**
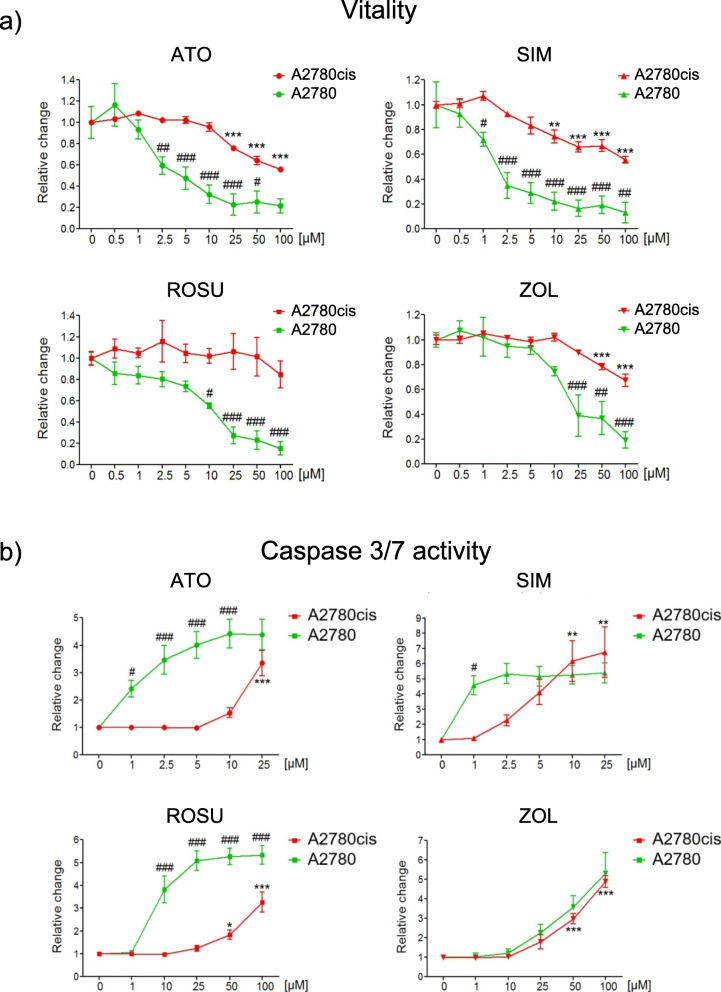
Cisplatin-resistant A2780cis cells show relative resistance to statins and zoledronic acid (ZOL) compared to parental A2780 cells. A2780 and A2780cis cells were treated with increasing concentrations of atorvastatin (ATO), simvastatin (SIM), rosuvastatin (ROSU) or (ZOL) for 48 h. Cell vitality (**a**) and apoptosis (**b**) were assessed by CellTiterBlue® and Caspase 3/7 Glo® assays. Statistics refers to A2780cis cells. Data are shown as mean ± SEM of at least three individual experiments. (*p < 0.05; **p < 0.01; ***p < 0.001 vs. respective control (0 μM). #*p* < 0.05; ##*p* < 0.01; ###*p* < 0.001 vs. respective value of A2780 treated cells)

One of the most challenging limitations in treating human ovarian cancer is the resistance of cancer cells to platinum [22]. Therefore, the anti-tumor effects mediated by statins and zoledronic acid were analyzed in the cisplatin-resistant cell line A2780cis, in direct comparison with the parental cisplatin-sensitive A2780 cell line. The relative cisplatin resistance of A2780cis cells was confirmed by showing that cisplatin affected vitality and apoptosis at higher doses, whereas parental A2780 cell vitality was already suppressed at low concentrations (Suppl. Fig. 4a). As previously demonstrated, the treatment of A2780 cells with mevalonate pathway inhibitors suppressed cell vitality (− 90%; Fig. 6a; *p* < 0.001) and induced caspase 3/7 activation by up to 5-folds (Fig. 6b; *p* < 0.001). Simvastatin and atorvastatin were more potent compared to rosuvastatin and zoledronic acid as significant anti-tumor effects were achieved at low concentrations (1–2.5 μM). A2780cis cells showed relative resistance to low concentrations of simvastatin, atorvastatin, and zoledronic acid (Fig. 6). Here, vitality was significantly decreased only at higher concentrations from 10 to 100 μM (− 30–40%; Fig. 6a; *p* < 0.001). These effects differed significantly from the loss of vitality achieved in A2780 cells that was attained already at lower concentrations (1–10 μM; *p* < 0.001). Rosuvastatin failed to induce significant effects on cell vitality in A2780cis cells. For caspase 3/7 activation, high concentrations of rosuvastatin and atorvastatin (25–100 μM) induced a 3-folds increase of apoptosis in A27080cis cells (Fig. 6b; *p* < 0.001). Notably, although higher concentrations (10–25 μM) were required, simvastatin induced a similar induction of caspases 3/7 activity in A2780cis cells compared to A2780 cells (6-folds; Fig. 6b; *p* < 0.01). These observations were confirmed by detection of cPARP showing a dose-dependent induction of apoptosis, which was accompanied by a significant suppression of *Svv* gene expression in A2780cis cells (− 80%; Suppl. Fig. 4b; *p* < 0.001). Here, simvastatin was able to block the mevalonate pathway even at concentrations as low as 1 μM (Suppl. Fig. 4b). Moreover, the pattern of caspase 3/7 activation upon treatment with zoledronic acid was similar between cisplatin-resistant and parental cells (6-folds; Fig. 6b; *p* < 0.01). These results demonstrate that cisplatin-resistant A2780cis cells show relative resistance to mevalonate pathway inhibitors, but respond with a similar induction of significant apoptosis compared to cisplatin-sensitive A2780 cells upon treatment with simvastatin and zoledronic acid.

## Discussion

Among human gynecologic malignancies, ovarian cancer is the most lethal one [56]. The vast majority of patients are diagnosed at an advanced stage, when the long term survival rates are found to be as low as 20–30% [5]. Current therapeutic options are limited, particularly for advanced ovarian cancer, because of recurrence, metastasis and chemo-resistance to primary treatments or secondarily acquired resistance [10]. Statins and N-BP as inhibitors of the mevalonate pathway have been widely studied for their pleiotropic anti-tumor properties [24].

The rationale for targeting the mevalonate pathway in ovarian cancer is underpinned by the evidence of a role of the mevalonate pathway in ovarian cancer tumorigenesis. The HMGCR enzyme is overexpressed in several human ovarian cancer cell lines, including IGROV-1 and A2780 [29]. Moreover, late-stage metastatic ovarian cancer cells show a marked molecular reprogramming with upregulation of several mevalonate pathway genes including HMGCR [57]. High levels of cholesterol have been linked to an increased risk of developing ovarian and other cancer entities [33]. A number of retrospective and case-control studies have shown a significant association between pre- and/or post-diagnostic use of statins and a reduced overall as well as ovarian cancer-specific mortality have been described [58, 59]. A reduced risk of developing ovarian or endometrial cancers was seen in patients prescribed with bisphosphonates for more than 1 year before the diagnosis [60].

In our study, we demonstrate that statins and the N-BP zoledronic acid reduce cell vitality and induce apoptosis in the ovarian cancer cell lines A2780 and IGROV-1. These observations match with studies describing similar effects in vitro and in vivo in a number of human malignancies [57, 61–72]. In the present study, the lipophilic statins atorvastatin and simvastatin were more potent than the hydrophilic rosuvastatin which is in line with studies in breast and additional ovarian cancer cell lines, including Hey 1B and OVCAR-3 [52, 63, 73–75]. The differences in the chemical structure, the transport into cells via passive membrane diffusion or via transporters and the varying potential to alter the sterol metabolism within tumor cells may account for variances in the anti-tumor effects exerted by different statins [41, 63].

Not only did the individual effects among the used statins differ, but also the sensitivity of the used cell lines, which is a known phenomenon in human cancer cell lines [52, 76, 77]. This could be due to differences in the molecular profile of A2780 and IGROV-1 cells. Although both of them are estrogen receptor negative, A2780 cells carry few mutations, whereas IGROV-1 are considered as hyper-mutated and carry both familial and sporadic mutations [78]. Moreover, simvastatin exerted significant effects on apoptosis in cisplatin-resistant A2780cis cells, although higher concentrations compared to parental A2780 cells were necessary, indicating a relative cross-resistance. Future studies might also investigate potential statin resistance mechanisms and biomarkers of statin or N-BP sensitivity and whether these may cross-interfere with mechanisms of cisplatin resistance. We and others have demonstrated a restorative feedback loop by induction of the statin targeting enzyme, the HMGCR, as a mediator of statin resistance in human breast and prostate cancer as well as multiple myeloma cells [52, 79, 80]. Similar mechanisms might occur in ovarian cancer cells, especially as cisplatin and simvastatin induce a strong accumulation of HMGCR in ovarian cancer cells [81, 82]. In times of personalized medicine, it would be greatly relevant to identify patients with those molecular subtypes, that would mostly benefit from a statin or N-BP therapy, individually or in combination with chemotherapy, as well as to define the optimal therapeutic window.

We demonstrated that the cytotoxic effects by statins and zoledronic acid in IGROV-1 and A2780 cells were mediated by an inhibited geranylgeranylation, rather than by an inhibited farnesylation. These observations were made in breast cancer cells using the same agents [51]. In mice bearing Ovcar-4 ovarian cancer xenografts, geranylgeraniol limits the anti-tumor effects of pitavastatin [29]. The underlying mechanisms are potentially mediated by a disrupted function of geranylgeranylated Rho proteins. Alendronate inhibits ovarian cancer cell migration through an inhibited geranylgeranylation and Rho protein activation [83]. In addition, pitavastatin alters the subcellular localization of RhoA, CDC42 and Ras in several ovarian cancer cell lines [77].

The treatments with statins and zoledronic acid also significantly reduced the expression of the anti-apoptotic genes *Bcl-2* and *Svv*, even in chemo-resistant A2780cis cells, which is in line with studies in breast, colorectal, and prostate cancer [84–87]. *Bcl-2* as well as *Svv* have been shown to be overexpressed in ovarian carcinomas and are associated with chemo-resistance and a worse prognosis [88–92]. The suppression of both genes by statins and zoledronic acid offers a potential strategy to overcome such resistance. Platinum resistance is a major problem in the treatment of ovarian cancer, for which different mechanisms account such as increased drug efflux and inactivation, the ability to repair platinum-DNA adducts, or overexpression of anti-apoptotic regulators [93]. Additional studies might investigate, as to whether or not statins and N-BP modulate anti-apoptotic genes in these cells and if a concomitant treatment with platinum would re-sensitize the cells and induce cytotoxicity as seen in cisplatin-resistant SKOV3 cells [94]. The potential advantage of such a combinatory approach is the reduction of individual concentrations without the loss or even with the increase of therapeutic effects and reduced side effects such as nephro- and neurotoxicity by cisplatin [11].

Our study also demonstrates, that especially statins significantly suppressed *TNF-α*, *TFG-β1 IL-6*, *IL-8* and *VEGF* in IGROV-1 and A2780 cells. These factors play central roles in the accumulation of peritoneal fluid, inflammation, and angiogenesis, as well as in supporting tumor promotion and metastases [54, 95–101]. Moreover, all of them are overexpressed and associated with chemo-resistance and a poor prognosis in affected patients with ovarian cancer [54, 95–101]. The constitutive expression of *IL6* by ovarian cancer cells stimulates TAMs of the tumor microenvironment thereby promoting tumor growth [102]. IL-6 also promotes the production of pro-angiogenic factors, like VEGF and IL-8 [96] and the latter itself drives cell motility [103]. Constitutive secretion of TNF-α leads to the further release of IL-6, VEGF, and other factors that concomitantly sustain the vicious cycle of malignant ascites [9, 54]. Targeting these tumor-promoting soluble factors offers a therapeutic strategy in ovarian cancer and might modulate the response of chemo-resistant tumor cells to chemotherapy. VEGF suppression was seen to reduce tumor burden and ascites formation in several preclinical studies [104]. Successful translation of these findings has led to the development and evaluation of the VEGF targeting antibody bevacizumab in clinical trials and its approval for the use in patients with ovarian cancer [13, 14]. Pharmacological inhibition of IL-6 has also been positively evaluated in preclinical and clinical studies [105]. The knockdown of IL-6 and IL-8 was shown to sensitize the tumor cells to cisplatin treatment in lung and ovarian cancer, respectively [106, 107]. Additional in vivo models using chemo-sensitive and -resistant ovarian cancer cell lines are necessary to evaluate the effects of statins and zoledronic acid, individually and in combination with chemotherapy, on these pro-tumorigenic factors.

Our study has several limitations, including the use of in vitro cell models which do not fully reflect the heterogenous pattern of molecular subtypes of human ovarian cancer. Moreover, the levels of statins that are reached in the serum of patients range between 0.002 and 0.1 μM [108] and the maximum concentration of zoledronic acid administered to patients is 2 μM [109]. Hence, the clinically achievable concentrations of these agents within tumor tissues and ascites might be too low for direct antitumor effects. However, the clonogenic assay revealed that even low concentrations of simvastatin significantly impaired the number of newly formed IGROV-1 colonies as a marker of the clonogenic potential. These findings might translate into effective anti-tumor effects of statins or even N-BP at low levels in the stage of single tumor cell dissemination to secondary sites and needs to be comprehensively analyzed in additional studies. In breast cancer, disseminated tumor cells can be eliminated with N-BP therapy and this is associated with an improved survival [110, 111]. For ovarian cancer, no trials exist that investigated adjuvant N-BP therapy in the context of single tumor cell dissemination.

## Conclusions

In conclusion, our study demonstrates pleiotropic anti-tumor effects in ovarian cancer cells in vitro, ranging from loss of vitality, induction of apoptosis and suppression of mediators implicated in tumor cell survival, migration, angiogenesis, and metastasis. Additional preclinical and clinical studies to evaluate the full anti-tumor potential of statins and zoledronic acid are highly warranted.

## Supplementary information

**Additional file 1: Suppl. Fig. 1.** The colony-forming ability of IGROV1 cells is significantly impaired by simvastatin (SIM). IGROV1 cells were seeded in 6-well plates (200 cells/cavity) and treated with atorvastatin (ATO), SIM, rosuvastatin (ROSU) or zoledronic acid (ZOL) for 9-10 days. The resulting colonies were stained with a crystal violet solution. **a.** The crystal violet staining was eluted with 10% SDS and the absorbance was measured at 595 nm. **b.** Counting of the colonies (absolute numbers). **c.** Representative pictures of the colonies. Data are shown as mean ± SEM of at least three individual experiments. *p < 0.05 vs. respective control (C).

**Additional file 2: Suppl. Fig. 2.** Statins and zoledronic acid (ZOL) do not modulate the expression of *B-cell lymphoma 2 (BCL-2),* but downregulate *survivin (SVV)* in A2780 cells. A2780 cells were treated with increasing concentrations of atorvastatin (ATO), simvastatin (SIM), rosuvastatin (ROSU) or ZOL for 24 h. Expression of *BCL-2* and *SVV* was assessed by real-time-PCR. Data are shown as mean ± SEM of at least three individual experiments. *p < 0.05; **p < 0.01; ***p < 0.001 vs. respective control (0 μM).

**Additional file 3: Suppl. Fig. 3.** Farnesyl pyrophosphate (FPP) and geranylgeranyl pyrophosphate (GGPP) specifically rescue farnesylation or geranylgeranylation and vitality upon mevalonate pathway inhibition in IGROV1 and A2780 cells. **a.** IGROV1 cells were treated with simvastatin (SIM; 10 μM) or zoledronic acid (ZOL; 50 μM), and supplemented with either FPP (50 μM) or GGPP (50 μM). Farnesylation of Ras, geranylgeranylation of Rap1A and cleavage of poly (ADP-ribose) polymerase (cPARP) were assessed by western blotting. Glyceraldehyde-3-phosphate dehydrogenase (GAPDH) was used as loading control. The figures show representative blots which were cropped from original images. Full-length blots are presented in Suppl. Fig. 7. Images were detected using GelCapture 7.0.18 software. **b.** A2780 cells were treated with atorvastatin (ATO), SIM, rosuvastatin (ROSU) or ZOL and supplemented with 10 μM of either FPP or GGPP for 48 h. Cell vitality was assessed by CellTiterBlue® assay. Data are shown as mean ± SEM of at least three individual experiments. *p < 0.05; **p < 0.01; ***p < 0.001 vs. respective control (C). #*p* < 0.05; ##*p* < 0.01; ###*p* < 0.001 vs. respective treatment (-).

**Additional file 4: Suppl. Fig. 4.** A2780CIS are relative resistant to cisplatin and undergo apoptosis upon mevalonate pathway inhibition with simvastatin (SIM). **a.** A2780 and A2780CIS cells were treated with increasing concentrations of cisplatin. Cell vitality was assessed by CellTiterBlue® assay (left axis), whereas apoptosis was assessed by Caspase 3/7 Glo® assay (right axis). Data are shown as mean ± standard deviation of at least three individual experiments. **b.** A2780CIS cells were treated with increasing concentrations of SIM for 48 h. Farnesylation of Ras, geranylgeranylation of Rap1a, and cleavage of poly (ADP-ribose) polymerase (cPARP) were assessed by western blotting. Glyceraldehyde-3-phosphate dehydrogenase (GAPDH) was used as loading control. The figures show representative blots which were cropped from original images. Full-length blots are presented in Suppl. Fig. 8. Images were detected using GelCapture 7.0.18 software. Expression of *SVV* was assessed by real-time-PCR. Data are shown as mean ± SEM of at least three individual experiments. **p < 0.01; ***p < 0.001 vs. respective control (0 μM).

**Additional file 5: Suppl. Fig. 5.** Uncropped Western Blots for Fig. 1a. The figure shows all original uncropped blots. As some membranes were used to simultaneously detect Ras and cleaved PARP (after cutting), the pictures here also include the cleaved PARP original blots used for Fig. 2a to keep the originality. All original blots for GAPDH are also included. Representative cropped GAPDH images are shown in Fig. 1a.

**Additional file 6: Suppl. Fig. 6.** Uncropped Western Blots for Fig. 2a. The figure shows all original uncropped blots. As some membranes were used to simultaneously detect Ras and cleaved PARP (after cutting), the pictures here also include the Ras original blots used for Fig. 1a to keep the originality. All original blots for GAPDH are also included. Representative cropped GAPDH images are shown in Fig. 2a.

**Additional file 7: Suppl. Fig. 7.** Uncropped Western Blots for Supplementary Figure 3a.

**Additional file 8: Suppl. Fig. 8.** Uncropped Western Blots for Supplementary Figure 4b.

## Data Availability

The datasets used and/or analyzed during the current study are available from the corresponding author on reasonable request.

## Abbreviations

ATO: Atorvastatin
BCL-2: B-cell lymphoma 2
cPARP: Cleaved PARP
DMSO: Dimethyl sulfoxide
ELISA: Enzyme-linked immunosorbent assay
FCS: Fetal calf serum
FPP: Farnesyl pyrophosphate
GAPDH: Glyceraldehyde 3-phosphate dehydrogenase
GGPP: Geranylgeranyl pyrophosphate
HMGCR: 3-hydroxy-3-methylglutaryl-CoA reductase
IL6: Interleukin 6
IL8: Interleukin 8
N-BP: Amino-bisphosphonates
PARP: Poly (ADP-ribose) polymerase
qRT-PCR: Quantitative real-time polymerase chain reaction
ROSU: Rosuvastatin
RT: Room temperature
SDS: Sodium dodecyl sulfate
SDS-PAGE: SDS polyacrylamide gel electrophoresis
SEM: Standard error of the mean
SIM: Simvastatin
SVV: Survivin
TAMs: Tumor-associated macrophages
TBS-T: Tris-buffered saline with 1% Tween-20
TGFβ1: Transforming growth factor β1
TNFA: Tumor necrosis factor α
VEGF: Vascular endothelial growth factor
ZOL: Zoledronic acid
