# Supplementary figures and images for: Anti-tumor effects of mevalonate pathway inhibition in ovarian cancer
Source: BMC Cancer. 2020 Jul 29;20:703. doi: 10.1186/s12885-020-07164-x (PMC7388525; doi:10.1186/s12885-020-07164-x)

## Slide 1
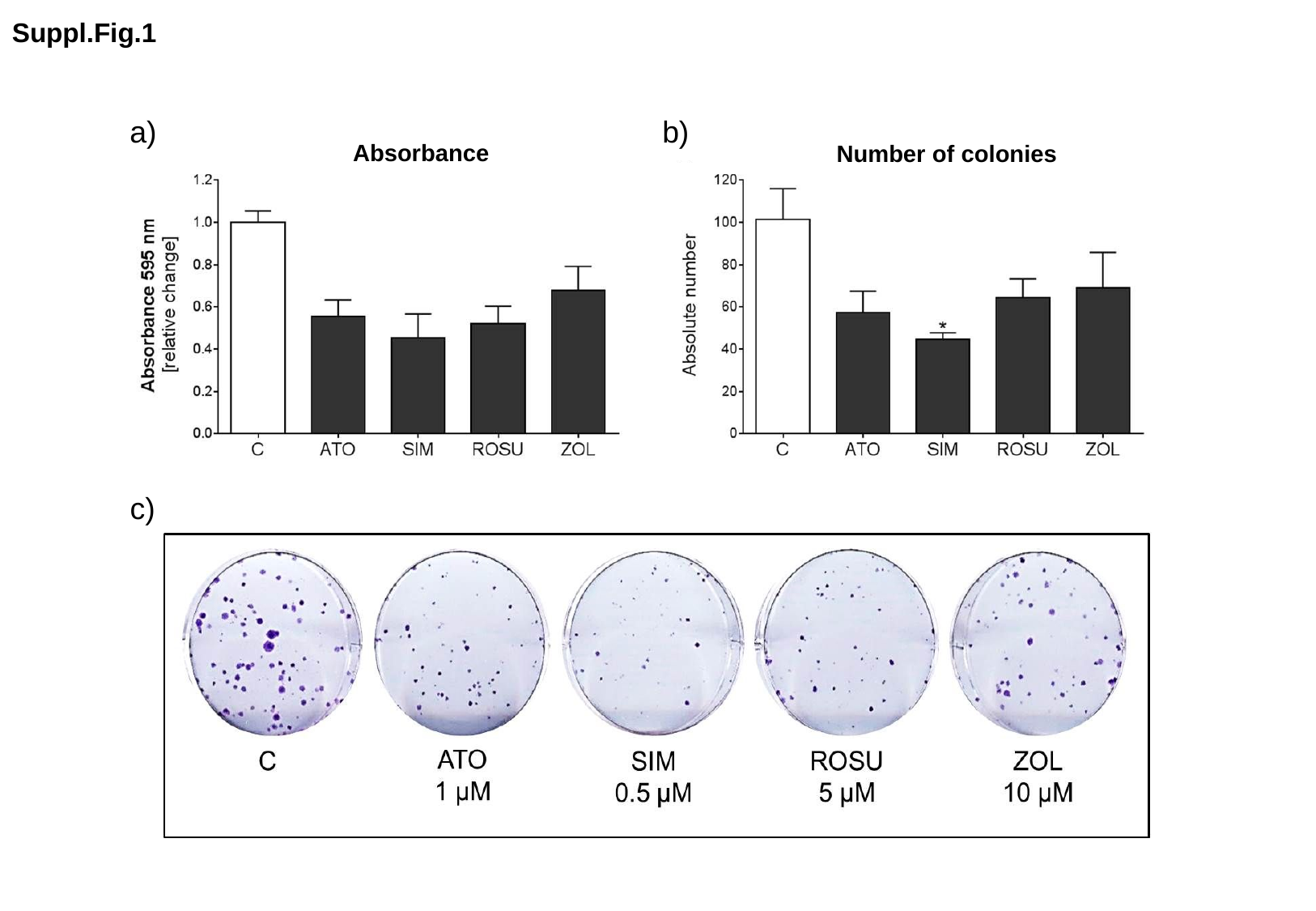

Suppl.Fig.1
a)
b)
Absorbance
Number of colonies
c)

Supplement: Supplementary file 1 — Additional file 1: Suppl. Fig. 1. The colony-forming ability of IGROV1 cells is significantly impaired by simvastatin (SIM). IGROV1 cells were seeded in 6-well plates (200 cells/cavity) and treated with atorvastatin (ATO), SIM, rosuvastatin (ROSU) or zoledronic acid (ZOL) for 9-10 days. The resulting colonies were stained with a crystal violet solution. a. The crystal violet staining was eluted with 10% SDS and the absorbance was measured at 595 nm. b. Counting of the colonies (absolute numbers). c. Representative pictures of the colonies. Data are shown as mean ± SEM of at least three individual experiments. *p < 0.05 vs. respective control (C). [file 12885_2020_7164_MOESM1_ESM.pptx]

## Slide 1
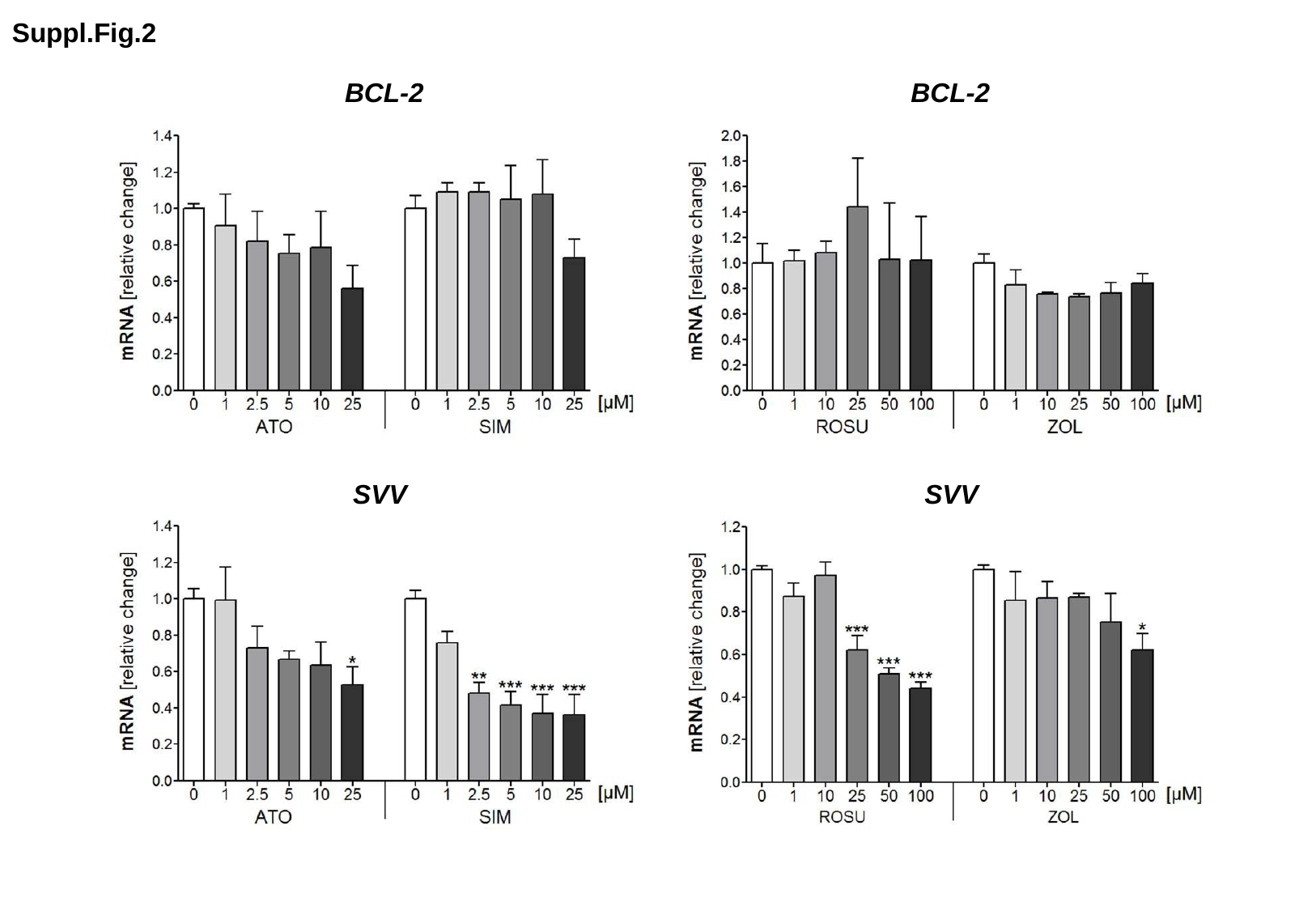

Suppl.Fig.2
BCL-2
BCL-2
SVV
SVV

Supplement: Supplementary file 2 — Additional file 2: Suppl. Fig. 2. Statins and zoledronic acid (ZOL) do not modulate the expression of B-cell lymphoma 2 (BCL-2), but downregulate survivin (SVV) in A2780 cells. A2780 cells were treated with increasing concentrations of atorvastatin (ATO), simvastatin (SIM), rosuvastatin (ROSU) or ZOL for 24 h. Expression of BCL-2 and SVV was assessed by real-time-PCR. Data are shown as mean ± SEM of at least three individual experiments. *p < 0.05; **p < 0.01; ***p < 0.001 vs. respective control (0 μM). [file 12885_2020_7164_MOESM2_ESM.pptx]

## Slide 1
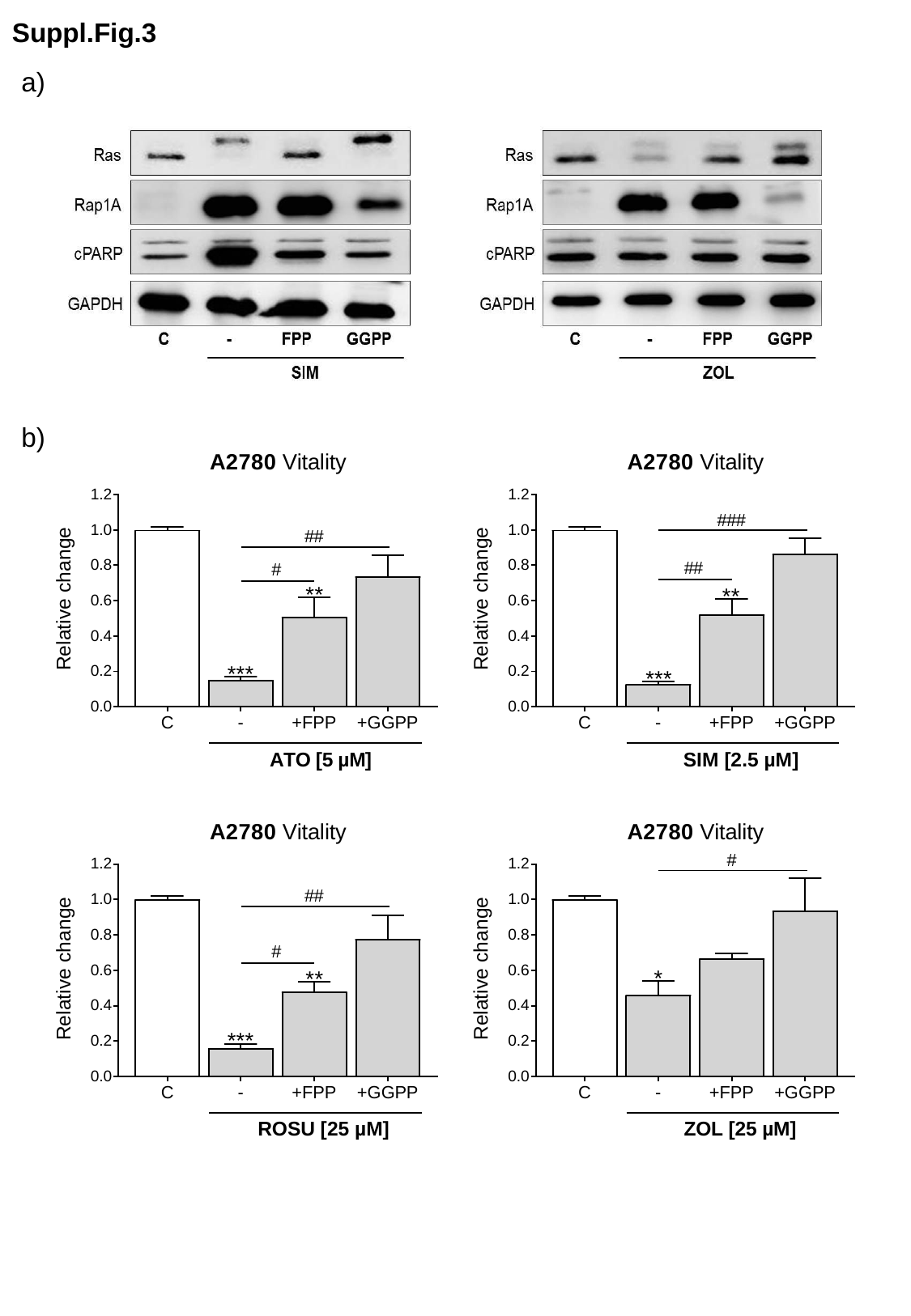

Suppl.Fig.3
a)
b)

Supplement: Supplementary file 3 — Additional file 3: Suppl. Fig. 3. Farnesyl pyrophosphate (FPP) and geranylgeranyl pyrophosphate (GGPP) specifically rescue farnesylation or geranylgeranylation and vitality upon mevalonate pathway inhibition in IGROV1 and A2780 cells. a. IGROV1 cells were treated with simvastatin (SIM; 10 μM) or zoledronic acid (ZOL; 50 μM), and supplemented with either FPP (50 μM) or GGPP (50 μM). Farnesylation of Ras, geranylgeranylation of Rap1A and cleavage of poly (ADP-ribose) polymerase (cPARP) were assessed by western blotting. Glyceraldehyde-3-phosphate dehydrogenase (GAPDH) was used as loading control. The figures show representative blots which were cropped from original images. Full-length blots are presented in Suppl. Fig. 7. Images were detected using GelCapture 7.0.18 software. b. A2780 cells were treated with atorvastatin (ATO), SIM, rosuvastatin (ROSU) or ZOL and supplemented with 10 μM of either FPP or GGPP for 48 h. Cell vitality was assessed by CellTiterBlue® assay. Data are shown as mean ± SEM of at least three individual experiments. *p < 0.05; **p < 0.01; ***p < 0.001 vs. respective control (C). #p < 0.05; ##p < 0.01; ###p < 0.001 vs. respective treatment (-). [file 12885_2020_7164_MOESM3_ESM.pptx]

## Slide 1
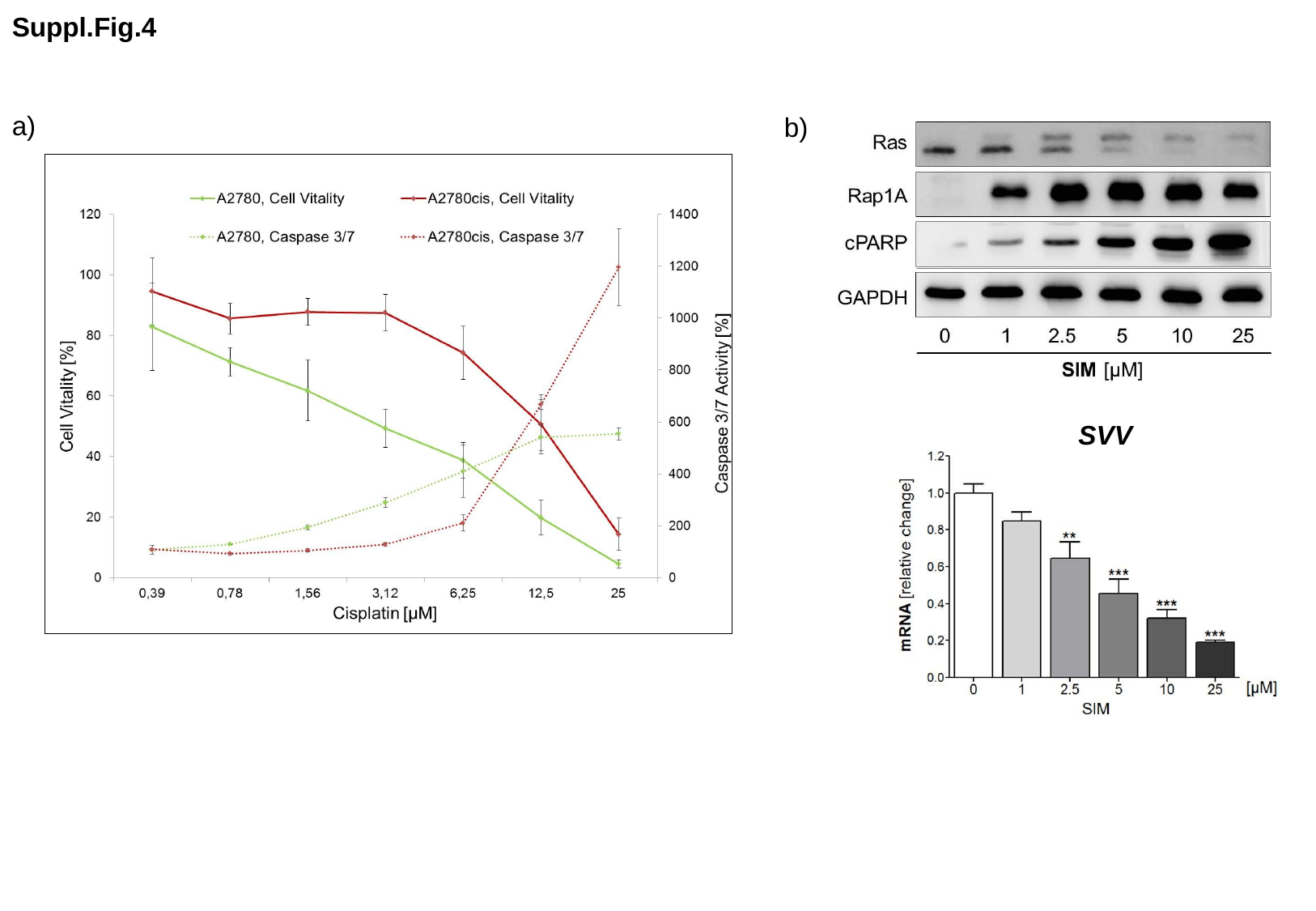

Suppl.Fig.4
a)
b)
SVV

Supplement: Supplementary file 4 — Additional file 4: Suppl. Fig. 4. A2780CIS are relative resistant to cisplatin and undergo apoptosis upon mevalonate pathway inhibition with simvastatin (SIM). a. A2780 and A2780CIS cells were treated with increasing concentrations of cisplatin. Cell vitality was assessed by CellTiterBlue® assay (left axis), whereas apoptosis was assessed by Caspase 3/7 Glo® assay (right axis). Data are shown as mean ± standard deviation of at least three individual experiments. b. A2780CIS cells were treated with increasing concentrations of SIM for 48 h. Farnesylation of Ras, geranylgeranylation of Rap1a, and cleavage of poly (ADP-ribose) polymerase (cPARP) were assessed by western blotting. Glyceraldehyde-3-phosphate dehydrogenase (GAPDH) was used as loading control. The figures show representative blots which were cropped from original images. Full-length blots are presented in Suppl. Fig. 8. Images were detected using GelCapture 7.0.18 software. Expression of SVV was assessed by real-time-PCR. Data are shown as mean ± SEM of at least three individual experiments. **p < 0.01; ***p < 0.001 vs. respective control (0 μM). [file 12885_2020_7164_MOESM4_ESM.pptx]

## Slide 1
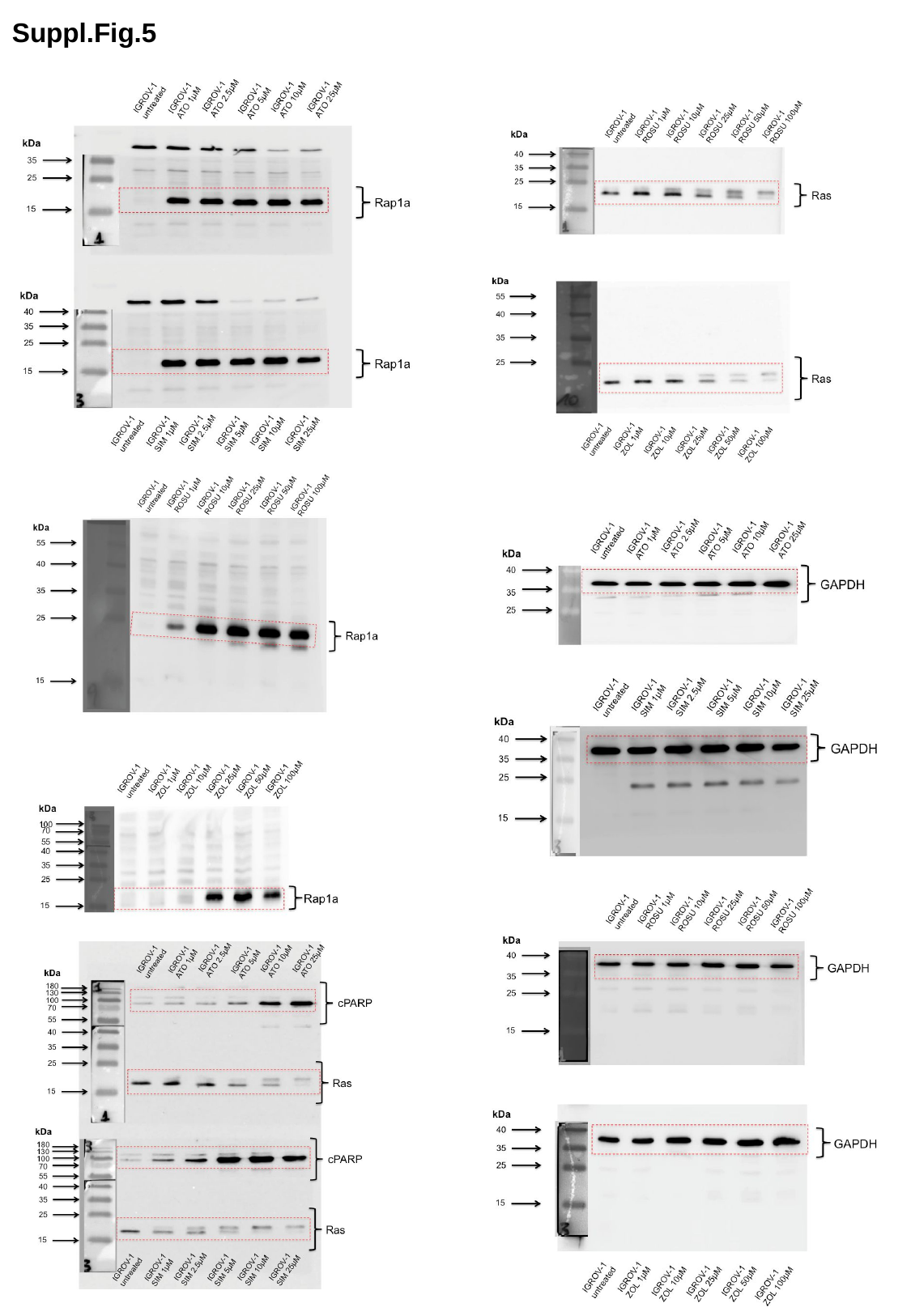

Suppl.Fig.5

## Slide 2
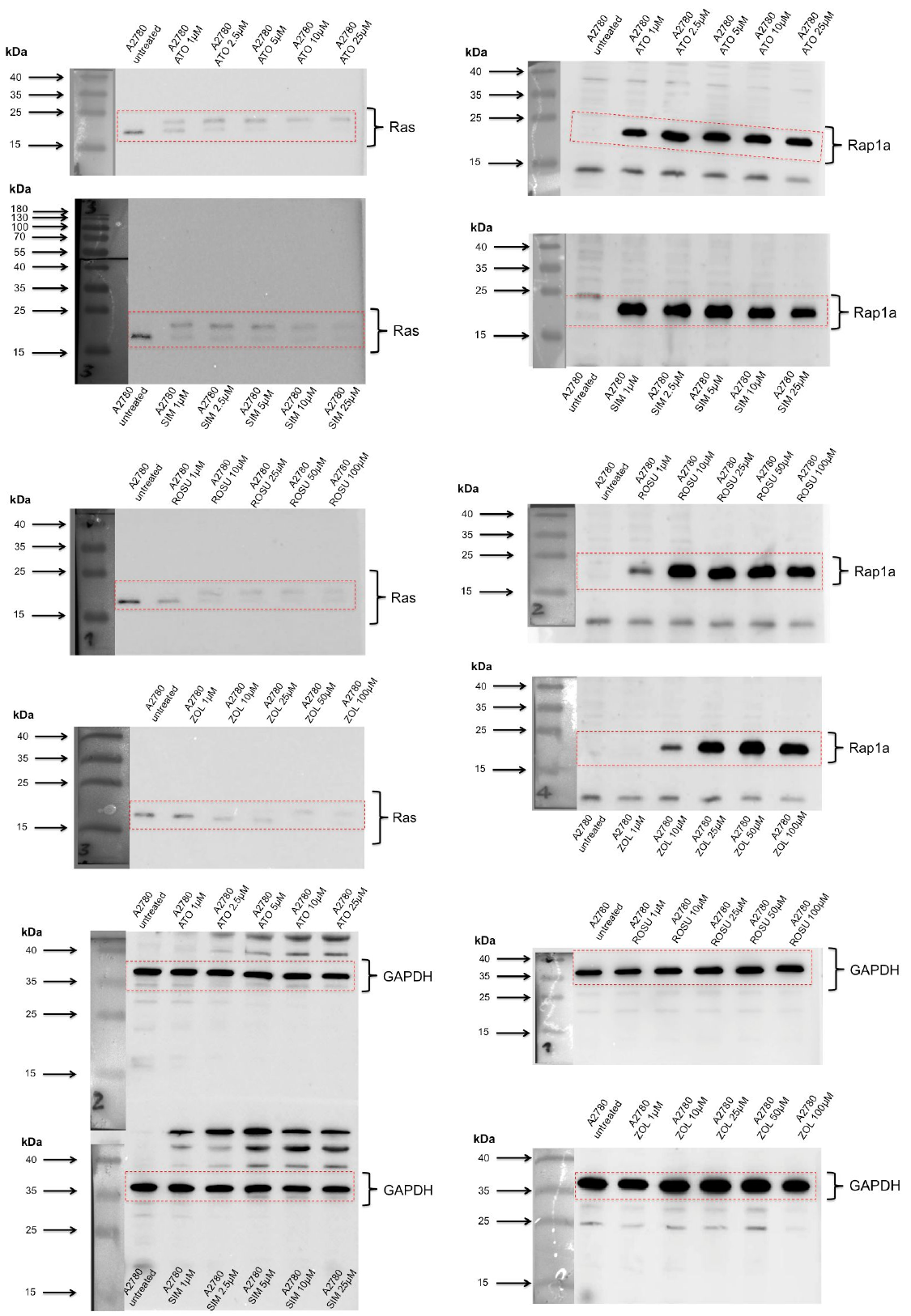

Supplement: Supplementary file 5 — Additional file 5: Suppl. Fig. 5. Uncropped Western Blots for Fig. 1a. The figure shows all original uncropped blots. As some membranes were used to simultaneously detect Ras and cleaved PARP (after cutting), the pictures here also include the cleaved PARP original blots used for Fig. 2a to keep the originality. All original blots for GAPDH are also included. Representative cropped GAPDH images are shown in Fig. 1a. [file 12885_2020_7164_MOESM5_ESM.pptx]

## Slide 1
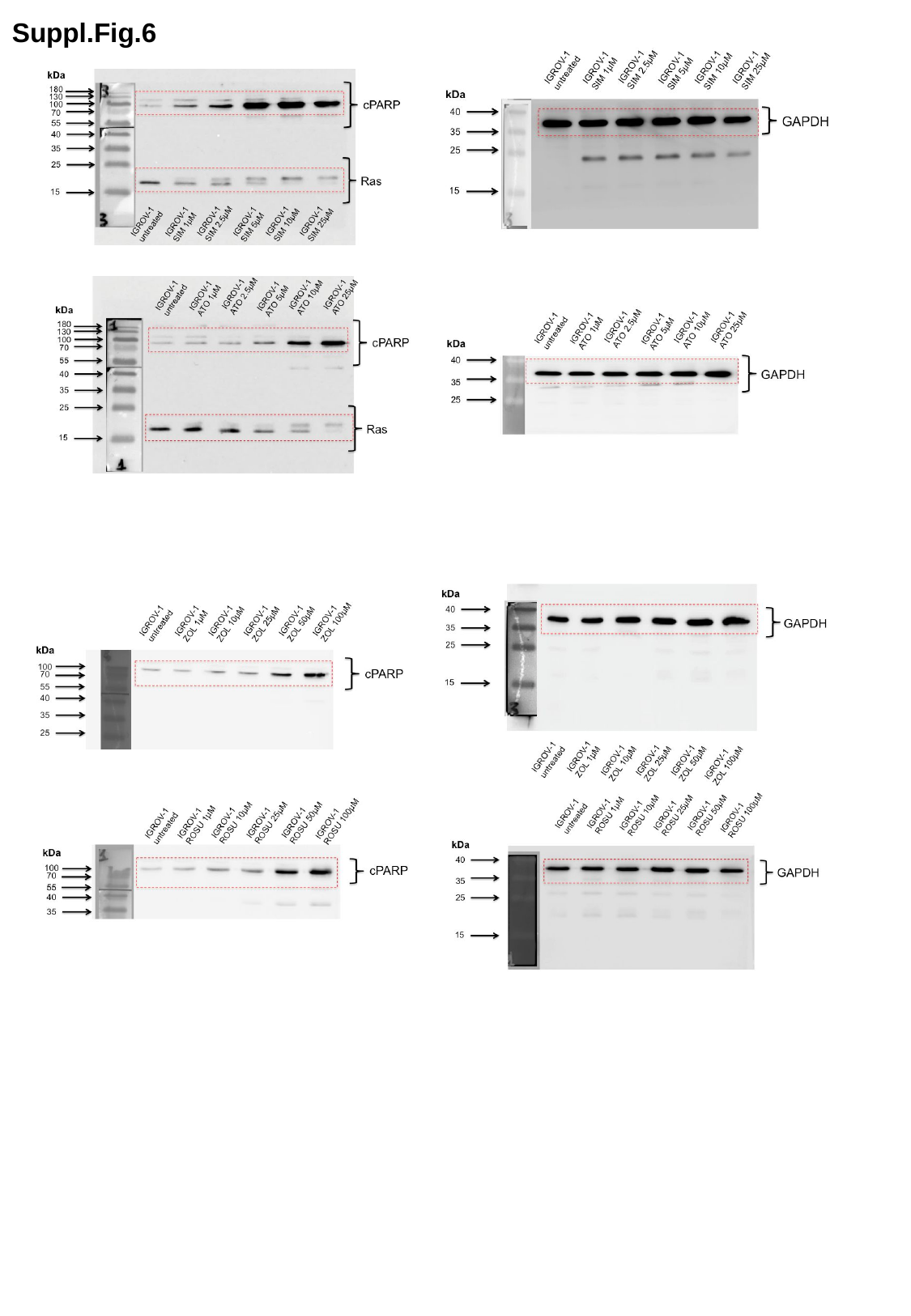

Suppl.Fig.6

## Slide 2
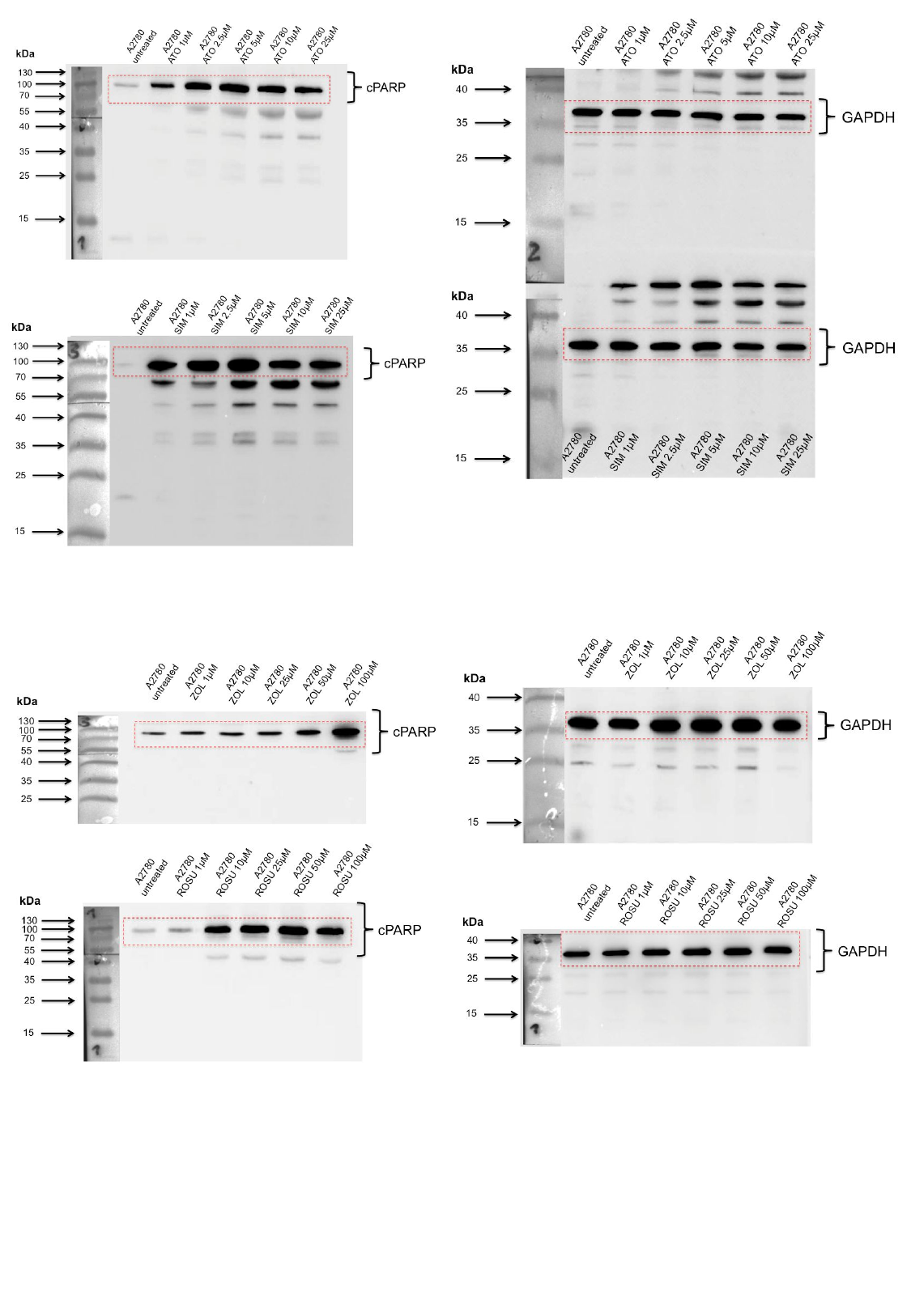

Supplement: Supplementary file 6 — Additional file 6: Suppl. Fig. 6. Uncropped Western Blots for Fig. 2a. The figure shows all original uncropped blots. As some membranes were used to simultaneously detect Ras and cleaved PARP (after cutting), the pictures here also include the Ras original blots used for Fig. 1a to keep the originality. All original blots for GAPDH are also included. Representative cropped GAPDH images are shown in Fig. 2a. [file 12885_2020_7164_MOESM6_ESM.pptx]

## Slide 1
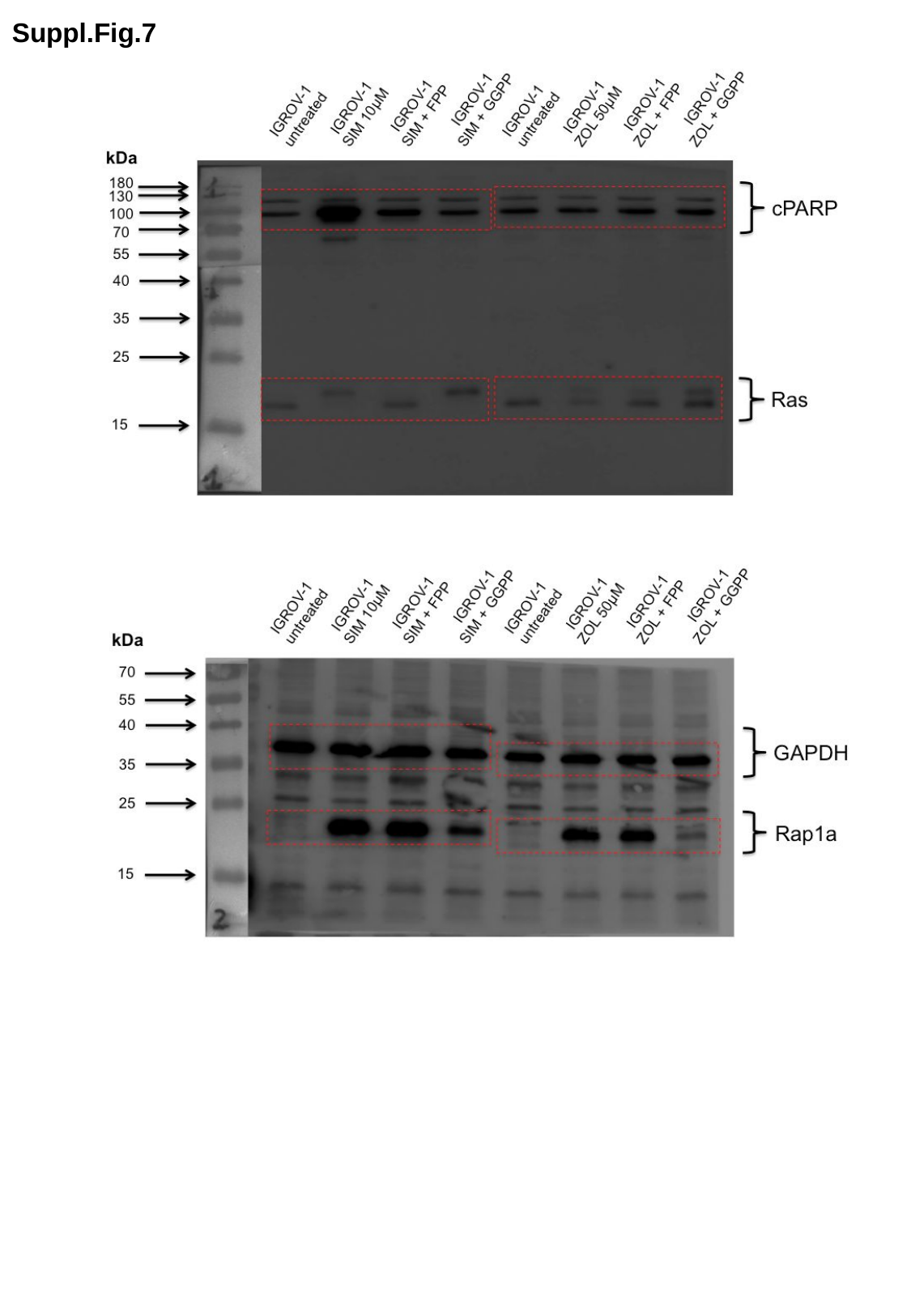

Suppl.Fig.7

Supplement: Supplementary file 7 — Additional file 7: Suppl. Fig. 7. Uncropped Western Blots for Supplementary Figure 3a. [file 12885_2020_7164_MOESM7_ESM.pptx]

## Slide 1
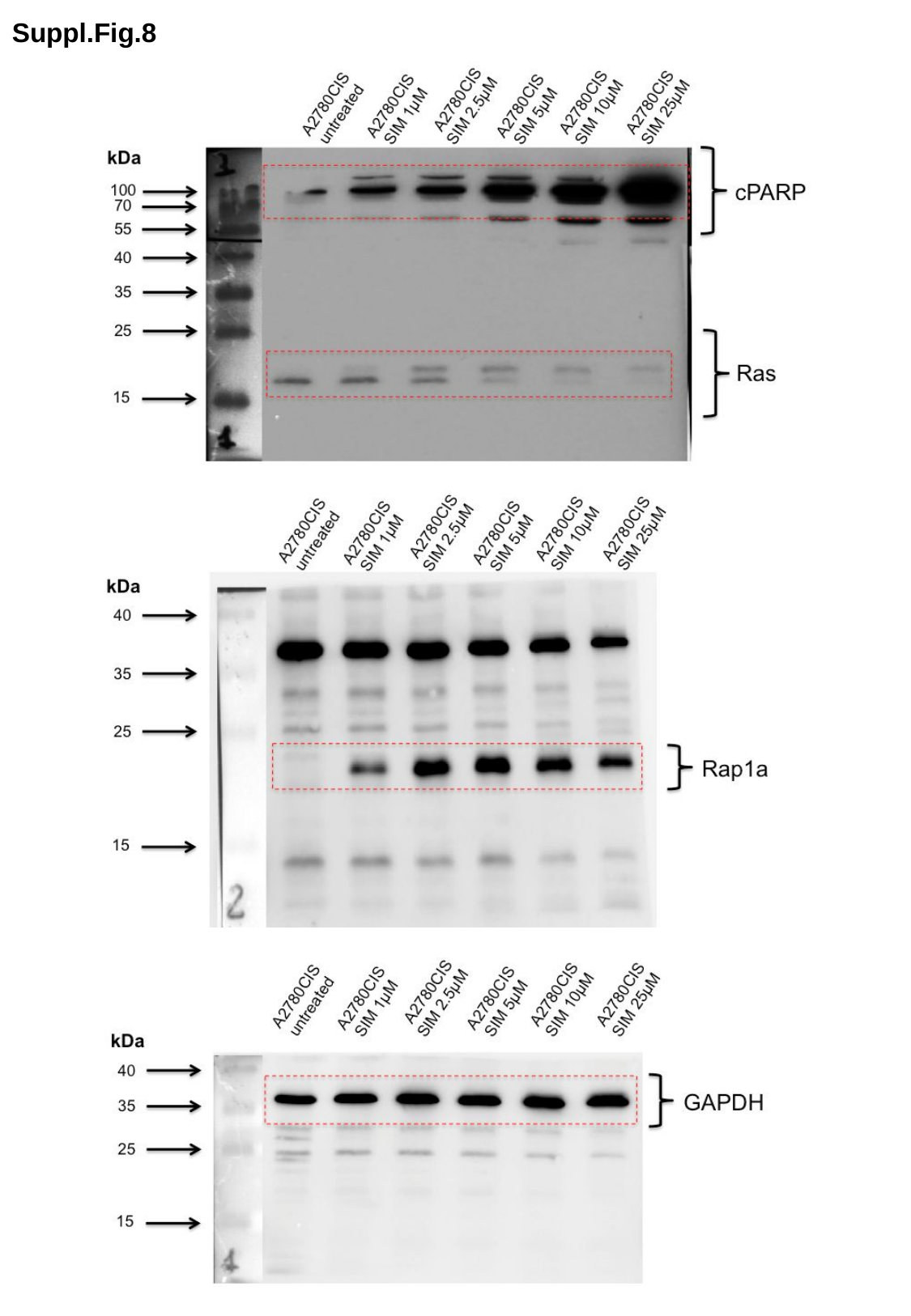

Suppl.Fig.8

Supplement: Supplementary file 8 — Additional file 8: Suppl. Fig. 8. Uncropped Western Blots for Supplementary Figure 4b. [file 12885_2020_7164_MOESM8_ESM.pptx]
